# Skeleton Synthesis of a Plant-Derived Radioprotective Alkaloid Born to Produce a Novel Fused Heterocycle

**DOI:** 10.3390/molecules28093829

**Published:** 2023-04-30

**Authors:** Sifan Liu, Huiling Gu, Kai Liang, Zhenzhen Wei, Bin Li, Ying Tian, Ruihong Li, Guangjie Zhang, Shuchen Liu

**Affiliations:** 1 Department of Pharmaceutical Science, Beijing Institute of Radiation Medicine, Beijing 100850, China; 18146539471@163.com (S.L.);; 2 School of Pharmacy, Henan University, Kaifeng 475001, China; 3 School of Pharmacy, Guangdong Pharmaceutical University, Guangzhou 510006, China; 4 Faculty of Environment and Life, Beijing University of Technology, Beijing 100022, China

**Keywords:** alkaloid, orychophragine D, novel molecular skeleton, chemical synthesis

## Abstract

Alkaloids are a material treasure bestowed on humans by nature owing to their numerous biological activities. Orychophragine D, an alkaloid isolated from the seeds of *Orychophragmus violaceus* was identified as bearing a novel skeleton and proved to have an excellent radioprotective effect. Different from the common alkaloid structure, the main block of orychophragine D is constructed of an oxotriazine and an oxopiperazine, which are connected in parallel by a C-N bond. In this paper, a preparation method for the novel heterocycle skeleton of orychophragine D is proposed for the first time. N-Boc-L-serine was utilized as the original material to complete the preparation with 11 steps in a 13% overall yield. A hydroxyl group was established on the side chain of the skeleton as the reaction site for researchers to conduct further structural modification or derivatization.

## 1. Introduction

Alkaloids are the weapons and shields of plants, protecting plants against biotic and abiotic stresses [1]. Biotic stress includes pests, pathogenic microorganisms, and predators. The main functions of alkaloids are to respond to antibacterial, antifungal, and antiherbivory biotic stress [2]. For example, piperine from black pepper and α- tomatine from tomatoes have been reported to have antibacterial and antifungal effects [3,4]. Swainsonine from Locoweed has been reported to cause intoxication in livestock due to its inhibition of α- mannosidase activity, affecting N-glycan cell membrane synthesis [5]. The abiotic pressures faced by plants mainly come from harsh environments such as drought, salinity, and high temperatures [6]. Alkaloids synthesized by plants are stored in organelles of specific glands, released, and exported to target tissues when stress signals from the environment are sensed [7,8]. In short, alkaloids are essential for plant survival.

**Figure 1:**
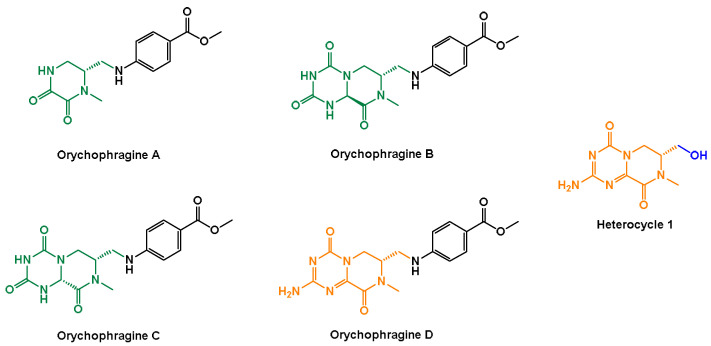
The structures of orychophragine A-D and the skeleton constructed by this study.

Alkaloids are also a material treasure bestowed on humans by nature owing to their numerous biological activities. Novel bioactive alkaloids from plants are constantly being discovered [9]. Alkaloids named orychophragine A-D isolated from the seeds of *Orychophragmus violaceus* have unique skeletons and excellent antiradiation or antitumor biological activity [10]. In one study, orychophragine D could improve the survival rate of mice to 100% at a radiation dose of 8 Gy, while the survival rate of the vehicle was 0% [11]. Different from the common alkaloid structure, the main block of orychophragine D consists of an oxotriazine and an oxopiperazine, which are connected in parallel by a C-N bond (Figure 1). In this study, a preparation method for this novel heterocycle skeleton of orychophragine D was developed for the first time. N-Boc- L-serine was used as the original material to prepare this heterocycle with 11 steps and a 13% overall yield. The hydroxyl group was established in the side chain of the fused heterocycle for researchers to conduct further transformation or derivatization of this heterocycle, which might lead to drug candidates with better radioprotective effects.

## 2. Results

**Scheme 1:**
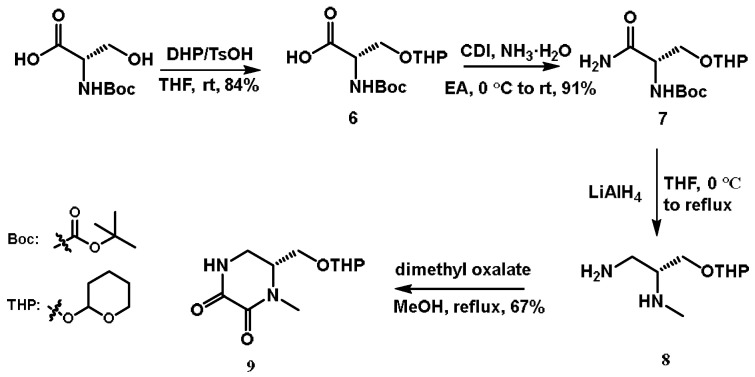
Synthesis of the dioxypiperazine intermediate.

We began our protocol with N-Boc-L-serine (Boc means t-butyloxy carbonyl, as shown in Scheme 1), with the chiral center being retained until the end product. The hydroxyl in **6** was protected by tetrahydropyran (THP) because THP can exist stably in the presence of a strong base or reducing agent during the synthesis process [12]. Carboxylic acid **6** was converted into an amide under the action of 1,1’-Carbonyldiimidazole (CDI) and ammonia to give **7**. Amide **7** was reduced with an excess dose of lithium aluminum hydride (LiAlH_4_) while the amide and N-Boc were reduced to an amino and a N-methyl, respectively, in a one-step reaction to give **8**. The Boc group on amino not only played a protective role, its reduced product, methyl, was also retained in the product as part of the backbone. Amine **8** underwent an ammonolysis with dimethyl oxalate to form dioxypiperazine **9**. 

**Scheme 2:**
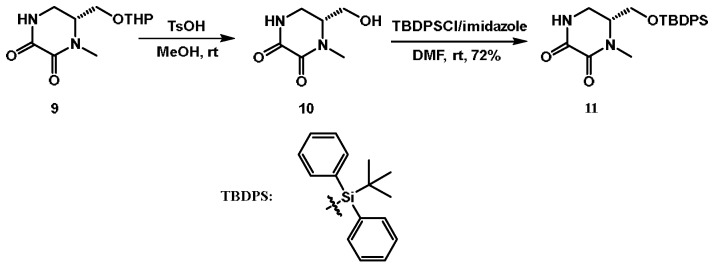
Replacement of the protecting group.

Benzyl (Bn) was initially used as the hydroxyl-protecting group in our protocol. Unfortunately, benzyl could not be removed in the synthesis route. Catalytic hydrogenation and other common methods to remove benzyl could not be performed. THP was selected as a qualified protective group due to its stability and ease of operation. However, THP could be selectively removed from the subsequently introduced Boc. We chose to replace the protective group in **9,** with tert-butyl diphenyl silyl (TBDPS) being a suitable alternative. TBDPS could be removed with tetrabutylammonium fluoride (TBAF), which has been proven to not affect the stability of Boc [13]. The removal of THP in **9** and the introduction of TBDPS in **10** were conducted following common methods described in the literature (Scheme 2). 

**Scheme 3:**
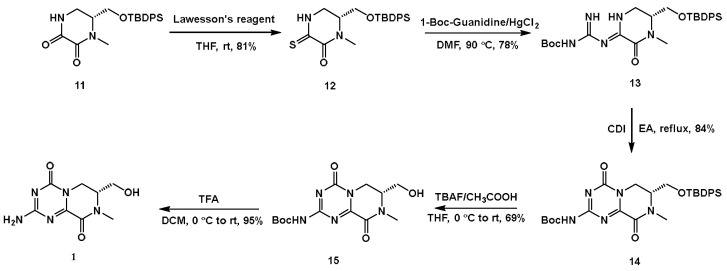
Skeleton construction of orychophragine D.

Two amide carbonyls in **11** (Scheme 3) were chemically selective for Lawesson reagent. No thiolation occurred on the carbonyl adjacent to the tertiary amine when the equivalent of the Lawesson reagent was controlled below 0.5, and sulfide **12** was therefore prepared efficiently. In the initial attempt to prepare **13**, we obtained a low yield because there were many factors that affected the reaction, such as the Lewis acid, solvent, temperature, and even the order in which the reactants were added. We found that if **12** and 1-Boc-guanidine were mixed before Lewis acid was added, this reaction had a better chemical selectivity. This addition order was applied to investigate the relationship between the reactants and yield (Table 1). We found that when mercuric chloride served as the Lewis acid and the solvent was N, N-dimethylformamide (DMF), **13** could be synthesized in a satisfactory yield. In the closed-loop reaction of **13**, CDI was proven to have a better performance than did triphosgene both in terms of operation and yield. The TBDPS and Boc in compound **14** were removed with TBAF and trifluoroacetic acid (TFA), respectively. The removal of TBDPS did not affect the stability of Boc to give **15**, which facilitated the further transformation and derivatization of this heterocycle.

## 3. Discussion

We exerted considerable effort in choosing and substituting the protecting groups in the synthesis route to achieve the chemoselectivity of reactions and selective removal of the protecting groups. A single exposed functional group could facilitate further modification or derivatization of the heterocycle by investigators. 

**Scheme 4:**
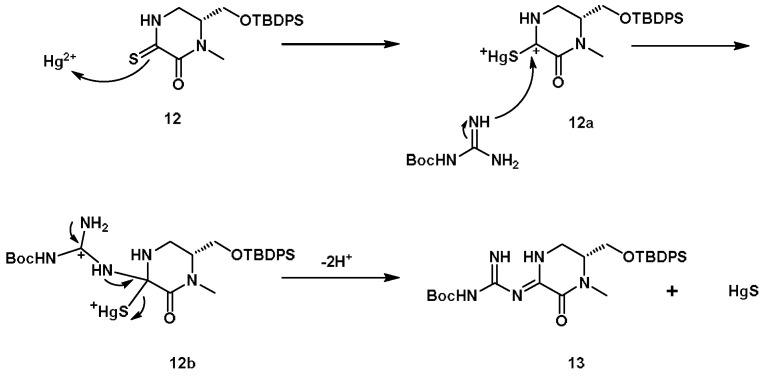
Possible mechanism for the preparation of 13.

During the preparation of **13**, it was found that the addition order of reactants had a significant impact on the yield. When **12** and Lewis acid were mixed in absence of 1-Boc-guanidine, many byproducts appeared; meanwhile, when **12** and 1-Boc-guanidine were mixed before Lewis acid was added, this reaction had a better chemical selectivity. According to this phenomenon, a prediction of the reaction mechanism was promoted, as shown in Scheme 4. When mercury chloride was mixed with **12** before 1-Boc-guanidine was added, the sulfur negative ions attacked the mercury ions to form a carbocation intermediate **12a**, and the carbocation ions of the intermediate **12a** attacked the negative electric groups within molecule **12**, resulting in the formation of by-products. If mercury chloride was added to the mixture of 1-Boc-guanidine and **12**, the intermediate **12a** preferentially attacked the more nucleophilic guanidine to form intermediate **12b**, in which the mercury sulfide left with a pair of electrons and two protons was removed with a base to form compound **13**.

## 4. Materials and Methods

### 4.1. Reagents and Instruments

All chemicals were obtained from a supplier (Sigma-Adrich, St. Louis, MO, USA, TCI, Ark). The NMR spectra were recorded with a JNM-ECA-400 spectrometer at 300K. Mass spectra were recorded with a Thermo Finnigan LCQ Advantage spectrometer. Silica gel chromatography was performed using 200–300 mesh silica gel.

### 4.2. Experimental Procedures

#### 4.2.1. N-(Tert-butoxycarbonyl)-O-(tetrahydro-2H-pyran-2-yl)-L-serine (**6**)

Compound **6** was prepared following the methods described in the literature [14].

#### 4.2.2. Tert-butyl((2S)-1-amino-1-oxo-3-((tetrahydro-2H-pyran-2-yl)oxy)propan-2-yl)carbamate (**7**)

Compound **6** (20 g, 69.4 mmol) was dissolved in EA (100 mL) in a 500 mL flask and cooled to 0 °C before CDI (13.4 g, 82.7 mmol, 1.2 eq) was added in batches with stirring. The mixture was stirred at rt for 2 h, and then 30% NH_3_·H_2_O (20 mL, 156 mmol, 2.2 eq) was added. It was stirred for another 2 h. The aqueous layer was extracted with EA (100 mL × 3), and the combined organic layer was washed with brine (100 mL), dried with Na_2_SO_4_, and concentrated in vacuo. The raw product was purified by column chromatography (CH_2_Cl_2_/MeOH 200:1 to 100:1) to yield a white solid (18.1 g, 91%). [α${]}_{D}^{24}$ − 18.5 (c 0.20, CH_3_OH). ^1^H NMR (400 MHz, DMSO-*d*_6_): *δ* 7.30 (d, *J* = 7.7 Hz, 1H), 7.07 (d, *J* = 9.3 Hz, 1H), 6.64 (dd, *J* = 24.0, 8.5 Hz, 1H), 4.52 (t, *J* = 3.2 Hz, 1H), 4.14–3.91 (m, 1H), 3.73–3.58 (m, 2H), 3.49–3.30 (m, 2H), 1.74–1.38 (m, 6H), and 1.34 (s, 9H). ^13^C NMR (101 MHz, DMSO-*d*_6_): *δ* 172.43, 172.30, 155.66, 98.60, 97.77, 78.62, 67.57, 67.30, 61.64, 61.50, 54.93, 54.45, 30.58, 30.49, 28.66, 25.50, 19.38, and 19.28. HRMS (ESI) *m*/*z* calcd for C_13_H_24_N_2_O_5_Na^+^ [M + Na]^+^: 311.1577, found: 311.1577.

#### 4.2.3. (2.R)-N2-Methyl-3-((tetrahydro-2H-pyran-2-yl)oxy)propane-1,2-diamine (**8**)

Compound 7 (18 g, 62.5 mmol) was dissolved in THF (200 mL) in a 1000 mL flask and cooled to 0 °C. LiAlH_4_ (9.2 g, 250 mmol, 4 eq) was slowly added in batches to form a suspension, which was refluxed at 80 °C for 24 h. The mixture was then cooled to 0 °C, and H_2_O (9 mL) and NaOH (20% in H_2_O, 9 mL) were successively added. The mixture was filtered, and the solvent of filtrate was removed in vacuo to obtain 16.2 g of raw product, which was used in the subsequent reaction without further purification.

#### 4.2.4. (6.R)-1-Methyl-6-(((tetrahydro-2H-pyran-2-yl)oxy)methyl)piperazine-2,3-dione (**9**)

Unpurified compound 8 (16.2 g) and dimethyl oxalate (14.7 g, 125 mmol, 2 eq) were mixed in MeOH (100 mL). The mixture was refluxed for 2 h. H_2_O (100 mL) was added, the aqueous layer was extracted with ethyl acetate (100 mL × 3), and the combined organic layer was washed with brine (100 mL), dried with Na_2_SO_4_, and concentrated in vacuo. The crude product was purified using column chromatography (CH_2_Cl_2_/MeOH 200:1 to 100:1) to obtain a white solid of 10.2 g. The two-step yield was 67%. [α${]}_{D}^{24}$ − 42.0 (c 0.20, CH_3_OH). ^1^H NMR (400 MHz, DMSO-*d*_6_): *δ* 8.37 (dd, *J* = 10.4, 5.1 Hz, 1H), 4.64–4.48 (m, 1H), 3.76–3.47 (m, 5H), 3.44–3.18 (m, 2H), 2.94 (d, *J* = 2.8 Hz, 3H), and 1.82–1.15 (m, 6H). ^13^C NMR (101 MHz, DMSO-*d*_6_): *δ* 158.28, 157.64, 98.68, 98.13, 66.07, 64.98, 61.62, 61.57, 56.30, 56.14, 34.22, 34.09, 30.51, 30.48, 25.45, and 19.19. HRMS (ESI) *m*/*z* calcd for C_11_H_19_N_2_O_4_^+^ [M + H]^+^: 243.1339, found: 243.1336.

#### 4.2.5. (R)-6-(Hydroxymethyl)-1-methylpiperazine-2,3-dione (**10**)

Compound 9 (1 g, 4.1 mmol) was mixed with p-toluene sulfonic acid (35 mg, 0.2 mmol, 0.05 eq) in 5 mL of methanol. The mixture was stirred for 2 h at rt. The solvent was removed in vacuo to yield 1.07 g of raw product, which was directly used in the next reaction without further purification.

#### 4.2.6. (R)-6-(((Tert-butyldiphenylsilyl)oxy)methyl)-1-methylpiperazine-2,3-dione (**11**)

Unpurified compound 10 (1 g) was mixed with imidazole (0.56 g, 8.3 mmol, 2 eq) in DMF (5 mL) before TBDPSCl (1.36 g, 4.9 mmol, 1.2 eq) was added. The mixture was stirred for 6 h at rt. H_2_O (10 mL) was added. The aqueous layer was extracted with EA (5 mL × 3), and the combined organic layer was washed with brine (10 mL × 3), dried with Na_2_SO_4_ and concentrated in vacuo. The crude product was purified using column chromatography (CH_2_Cl_2_/MeOH 200:1 to 100:1) and dried in vacuo to obtain a white solid of 1.18 g. The two-step yield was 72%. [α${]}_{D}^{24}$ − 35.5 (c 0.20, CH_3_OH). ^1^H NMR (400 MHz, DMSO-*d*_6_): *δ* 8.41 (d, *J* = 4.9 Hz, 1H), 7.60 (t, *J* = 7.8 Hz, 4H), 7.52–7.41 (m, 6H), 3.74–3.66 (m, 3H), 3.64 (dd, *J* = 10.8, 5.1 Hz, 1H), 3.37 (dd, *J* = 13.0, 5.3 Hz, 1H), and 2.89 (s, 3H). ^13^C NMR (101 MHz, DMSO-*d*_6_): *δ* 157.68, 157.00, 135.08, 135.06, 132.32, 130.04, 128.02, 62.03, 57.20, 38.96, 33.72, 26.53, and 18.65. HRMS (ESI) *m*/*z* calcd for C_22_H_29_N_2_O_3_Si^+^ [M + H]^+^: 397.1942, found: 397.1944.

#### 4.2.7. (R)-6-(((Tert-butyldiphenylsilyl)oxy)methyl)-1-methyl-3-thioxopiperazin-2-one (**12**)

Compound 11 (1 g, 2.5 mmol) was mixed with Lawesson reagent (0.51 g, 1.2 mmol, 0.5 eq) in THF (5 mL). The mixture was stirred for 6 h at rt. H_2_O (5 mL) was added. The aqueous layer was extracted with EA (5 mL × 3), and the combined organic layer was washed with brine (5 mL), dried with Na_2_SO_4_, and concentrated in vacuo. The crude product was purified using column chromatography (CH_2_Cl_2_/MeOH 200:1 to 100:1) and dried in vacuo to obtain a yellow solid of 842 mg. The yield was 81%. [α${]}_{D}^{24}$ − 60.5 (c 0.20, CH_3_OH).^1^H NMR (400 MHz, DMSO-*d*_6_): *δ* 10.94 (d, *J* = 4.9 Hz, 1H), 7.65–7.27 (m, 10H), 3.80–3.49 (m, 4H), 3.44–3.33 (m, 1H), 2.89 (s, 3H), and 0.95 (s, 9H). ^13^C NMR (101 MHz, DMSO-*d*_6_): *δ*: 186.09, 156.44, 135.58, 132.69, 130.56, 128.58, 62.40, 57.73, 41.81, 35.28, 27.10, and 19.18. HRMS (ESI) *m*/*z* calcd for C_22_H_29_N_2_O_2_SSi^+^ [M + H]^+^: 413.1714, found: 413.1714.

#### 4.2.8. Tert-butyl ((Z)-amino(((R,Z)-5-(((tert-butyldiphenylsilyl)oxy)methyl)-4-methyl-3-oxopiperazin-2-ylidene)amino)methylene)carbamate (**13**)

Compound 12 (410 mg, 1 mmol) was mixed with 1-Boc-guadine (0.32 g, 2 mmol, 2 eq) in DMF (3 mL). HgCl_2_ (297 mg, 1.1 mmol, 1,1 eq) was added, and the mixture was stirred for 2 h at 90 °C. H_2_O (5 mL) was added. The aqueous layer was extracted with EA (5 mL × 3), and the combined organic layer was washed with brine (5 mL × 3), dried with Na_2_SO_4_, and concentrated in vacuo. The crude product was purified using column chromatography (CH_2_Cl_2_/MeOH 200:1 to 100 solid 417 mg). The yield was 78%. (When replacing HgCl_2_ with equivalent Zn(OAc)_2_ as a safer Lewis acid, the yield was 70%.) [α${]}_{D}^{24}$ − 114.0 (c 0.20, CH_3_OH). ^1^H NMR (400 MHz, DMSO-*d*_6_): *δ* 9.06 (s, 1H), 8.10 (s, 1H), 7.59–7.52 (m, 4H), 7.48–7.40 (m, 6H), 3.96 (dd, *J* = 16.9, 6.4 Hz, 1H), 3.86 (d, *J* = 16.6 Hz, 1H), 3.77 (dd, *J* = 10.7, 4.5 Hz, 1H), 3.71–3.64 (m, 2H), 2.96 (s, 3H), 1.38 (s, 9H), and 0.93 (s, 9H). ^13^C NMR (101 MHz, DMSO-*d*_6_): *δ* 162.81, 158.29, 154.69, 147.85, 135.56, 132.74, 132.66, 130.50, 128.47, 77.40, 63.81, 56.82, 46.79, 36.30, 34.00, 31.27, 28.54, 26.92, and 19.04. HRMS (ESI) *m*/*z* calcd for C_28_H_40_N_5_O_4_Si^+^ [M + H]^+^: 538.2844, found: 538.2845.

#### 4.2.9. Tert-butyl (R)-(7-(((tert-butyldiphenylsilyl)oxy)methyl)-8-methyl-4,9-dioxo-6,7,8,9-tetrahydro-4H-pyrazino [1,2-a][1,3,5]triazin-2-yl)carbamate (**14**)

Compound 13 (537 mg, 1 mmol) was dissolved in EA (3 mL). CDI (178 mg, 1.1 mmol, 1.1 eq) was added, and the mixture was stirred for 3 h at reflux. It was cooled to rt before H_2_O (3 mL) was added. The aqueous layer was extracted using EA (3 mL × 3), and the combined organic layer was washed with brine (3 mL × 1), dried (Na_2_SO_4_), filtered, and concentrated in vacuo. The crude product was purified using column chromatography (CH_2_Cl_2_/MeOH 200:1 to 100:1) and dried in vacuo to obtain a white solid (472 mg). The yield was 84%. [α${]}_{D}^{24}$ − 48.0 (c 0.20, CH_3_OH). ^1^H NMR (400 MHz, DMSO-*d*_6_): *δ* 10.59 (s, 1H), 7.65–7.23 (m, 10H), 4.50 (d, *J* = 13.3 Hz, 1H), 4.10–3.86 (m, 2H), 3.70 (s, 2H), 2.92 (s, 3H), 1.39 (s, 9H), and 0.80 (s, 9H). ^13^C NMR (101 MHz, DMSO-*d*_6_): *δ* 164.03, 155.92, 155.66, 154.43, 150.17, 135.51, 135.38, 132.41, 132.36, 130.56, 130.51, 128.53, 128.47, 80.76, 63.99, 55.38, 42.33, 34.47, 28.24, 26.87, and 18.93. HRMS (ESI) *m*/*z* calcd for C29H38N5O5Si^+^ [M + H]^+^: 564.2637, found: 564.2641.

#### 4.2.10. Tert-butyl (R)-(7-(hydroxymethyl)-8-methyl-4,9-dioxo-6,7,8,9-tetrahydro-4H-pyrazino [1,2-a][1,3,5]triazin-2-yl)carbamate (**15**)

Compound 14 (100 mg, 178 μmol) was mixed with CH_3_COOH (20 μL, 355 μmol, 2 eq) in THF (2 mL). TBAF (1M in THF, 360 μL, 360 μmol, 2eq) was added dropwise at 0 °C, and the mixture was restored to rt and stirred for 1 h. The mixture was filtered, and the residue was washed with THF (2 mL × 2) and dried in vacuo to obtain a white solid of 40 mg with a yield of 69%. [α${]}_{D}^{24}$ − 14.0 (c 0.20, CH_3_OH). ^1^H NMR (400 MHz, DMSO-*d*_6_): *δ* 10.56 (s, 1H), 5.19 (s, 1H), 4.33 (d, *J* = 13.3 Hz, 1H), 3.89 (dd, *J* = 14.2, 5.3 Hz, 1H), 3.81 (d, *J* = 4.2 Hz, 1H), 3.62–3.54 (m, 2H), 3.06 (s, 3H), and 1.43 (s, 9H). ^13^C NMR (101 MHz, DMSO-*d*_6_): *δ* 163.47, 155.52, 155.45, 153.89, 149.68, 80.33, 60.19, 55.44, 41.80, 34.00, and 27.73. HRMS (ESI) *m*/*z* calcd for C_13_H_20_N_5_O_5_^+^ [M + H]^+^: 326.1459, found: 326.1445.

#### 4.2.11. (R)-2-Amino-7-(hydroxymethyl)-8-methyl-7,8-dihydro-4H-pyrazino [1,2-a][1,3,5]triazine-4,9(6H)-dione (**1**)

Compound 15 (1.5 g, 4.6 mmol) was dissolved in 1 mL of CH_2_Cl_2_. TFA (1 mL, 13.5 mmol, 3 eq) was added dropwise at 0 °C, and the mixture was stirred at 0 ° C for 30 min. The solvent was removed in vacuo to obtain a white solid of 993 mg with the following yield of 95%. [α${]}_{D}^{24}$ − 2.5 (c 0.20, H_2_O). ^1^H NMR (600 MHz, DMSO-*d_6_*): δ 7.89 (s, 1H), 7.61 (s, 1H), 5.15 (s, 1H), 4.32 (d, J = 12.6 Hz, 1H), 3.80–3.72 (m, 2H), 3.55 (d, J = 3.8 Hz, 2H), and 3.05 (s, 3H). ^13^C NMR (151 MHz, DMSO-*d_6_*): δ 165.61, 155.79, 154.20, 153.64, 60.06, 55.74, 40.89, and 34.08. HRMS (ESI) *m*/*z* calcd for C_8_H_12_N_5_O_3_^+^ [M + H]^+^: 226.0935, found: 226.0935.

## 5. Conclusions

The novel heterocycle skeleton in orychophragine D, a promising radioprotective alkaloid derived from the seeds of *Orychophragmus violaceus* was prepared. The synthesis was started with N-Boc-L-serine and completed in 11 steps and a 13% overall yield. The hydroxyl group was established on the side chain of the skeleton as the reaction site for researchers to conduct further structural modification or derivatization. We hope that this study could contribute to the discovery of new molecules with excellent radiation protective activity.

## Figures and Tables

**Table 1:** Reaction condition screening.

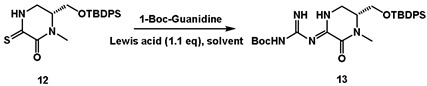			
Lewis Acid	Solvent	Temperature (°C)	Yield
ZnCl_2_	1.4-dioxane	25	NR *
ZnCl_2_	DMF	25	NR
ZnCl_2_	acetonitrile	25	NR
ZnCl_2_	toluene	25	NR
ZnCl_2_	1.4-dioxane	90	53%
ZnCl_2_	DMF	90	65%
ZnCl_2_	acetonitrile	90	16%
ZnCl_2_	toluene	90	39%
CuCl_2_	DMF	90	48%
CuCl_2_	1.4-dioxane	90	34%
CuCl_2_	toluene	90	20%
Zn(AcO)_2_	DMF	90	70%
Zn(AcO)_2_	1.4-dioxane	90	55%
Zn(AcO)_2_	toluene	90	42%
HgCl_2_	DMF	90	78%
HgCl_2_	1.4-dioxane	90	71%
HgCl_2_	toluene	90	40%

* NR—no reaction.

## Data Availability

The data presented in this study are available in the Supplementary Materials.

## Supplementary Materials

The following supporting information can be downloaded at https://www.mdpi.com/article/10.3390/molecules28093829/s1: 1 Figure S1: ^1^H-NMR spectrum of **7** in DMSO-*d*_6_. Figure S2: ^13^C-NMR spectrum of **7** in DMSO-*d*_6_. Figure S3: HR-ESI-MS spectrum of **7.** Figure S4: ^1^H-NMR spectrum of **9** in DMSO-*d*_6_. Figure S5: ^13^C-NMR spectrum of **9** in DMSO-*d*_6_. Figure S6: HR-ESI-MS spectrum of **9.** Figure S7: ^1^H-NMR spectrum of **11** in DMSO-*d*_6_. Figure S8: ^13^C-NMR spectrum of **11** in DMSO-*d*_6_. Figure S9: HR-ESI-MS spectrum of **11.** Figure S10: ^1^H-NMR spectrum of **12** in DMSO-*d*_6_. Figure S11: ^13^C-NMR spectrum of **12** in DMSO-*d*_6_. Figure S12: HR-ESI-MS spectrum of **12.** Figure S13: ^1^H-NMR spectrum of **13** in DMSO-*d*_6_. Figure S14: ^13^C-NMR spectrum of **13** in DMSO-*d*_6_. Figure S15: HR-ESI-MS spectrum of **13.** Figure S16: ^1^H-NMR spectrum of **14** in DMSO-*d*_6_. Figure S17: ^13^C-NMR spectrum of **14** in DMSO-*d*_6_. Figure S18: HR-ESI-MS spectrum of **14.** Figure S19: ^1^H-NMR spectrum of **15** in DMSO-*d*_6_.Figure S20: ^13^C-NMR spectrum of **15** in DMSO-*d*_6_. Figure S21: HR-ESI-MS spectrum of **15.** Figure S22. ^1^H-NMR spectrum of **1** in DMSO-*d*_6_. Figure S23. ^13^C-NMR spectrum of **1** in DMSO-*d*_6_. Figure S24. HR-ESI-MS spectrum of **1**. Figure S25. ^1^H-^1^H COSY spectrum of **1.** Figure S26. HSQC spectrum of **1.** Figure S27. HMBC spectrum of **1.**

## Sample Availability

Samples of the compounds are available from the authors.
