# Supplementary material for: Skeleton Synthesis of a Plant-Derived Radioprotective Alkaloid Born to Produce a Novel Fused Heterocycle
Source: Molecules. 2023 Apr 30;28(9):3829. doi: 10.3390/molecules28093829 (PMC10179810; doi:10.3390/molecules28093829)
Supplement: Supplementary file 1 [file molecules-28-03829-s001.zip › molecules-2340506-supplementary.pdf]

Supplementary Material

1 Supplementary Figure S1. ^{1}H-NMR spectrum of 7 in DMSO-d_6

2 Supplementary Figure S2. ^{13}C-NMR spectrum of 7 in DMSO-d_6

3 Supplementary Figure S3. HR-ESI-MS spectrum of 7

4 Supplementary Figure S4. ^{1}H-NMR spectrum of 9 in DMSO-d_6

5 Supplementary Figure S5. ^{13}C-NMR spectrum of 9 in DMSO-d_6

6 Supplementary Figure S6. HR-ESI-MS spectrum of 9

7 Supplementary Figure S7. ^{1}H-NMR spectrum of 11 in DMSO-d_6

8 Supplementary Figure S8. ^{13}C-NMR spectrum of 11 in DMSO-d_6

9 Supplementary Figure S9. HR-ESI-MS spectrum of 11

10 Supplementary Figure S10. ^{1}H-NMR spectrum of 12 in DMSO-d_6

11 Supplementary Figure S11. ^{13}C-NMR spectrum of 12 in DMSO-d_6

12 Supplementary Figure S12. HR-ESI-MS spectrum of 12

13 Supplementary Figure S13. ^{1}H-NMR spectrum of 13 in DMSO-d_6

14 Supplementary Figure S14. ^{13}C-NMR spectrum of 13 in DMSO-d_6

15 Supplementary Figure S15. HR-ESI-MS spectrum of 13

16 Supplementary Figure S16. ^{1}H-NMR spectrum of 14 in DMSO-d_6

17 Supplementary Figure S17. ^{13}C-NMR spectrum of 14 in DMSO-d_6

18 Supplementary Figure S18 HR-ESI-MS spectrum of 14

19 Supplementary Figure S19. ^{1}H-NMR spectrum of 15 in DMSO-d_6

20 Supplementary Figure S20. ^{13}C-NMR spectrum of 15 in DMSO-d_6

21 Supplementary Figure S21. HR-ESI-MS spectrum of 15

22 Supplementary Figure S22. ^{1}H-NMR spectrum of 1 in DMSO-d_6

23 Supplementary Figure S23. ^{13}C-NMR spectrum of 1 in DMSO-d_6

24 Supplementary Figure S24. HR-ESI-MS spectrum of 1

25 Supplementary Figure S25. ^{1}H-^{1}H COSY spectrum of 1

26 Supplementary Figure S26. HSQC spectrum of 1

27 Supplementary Figure S27. HMBC spectrum of 1

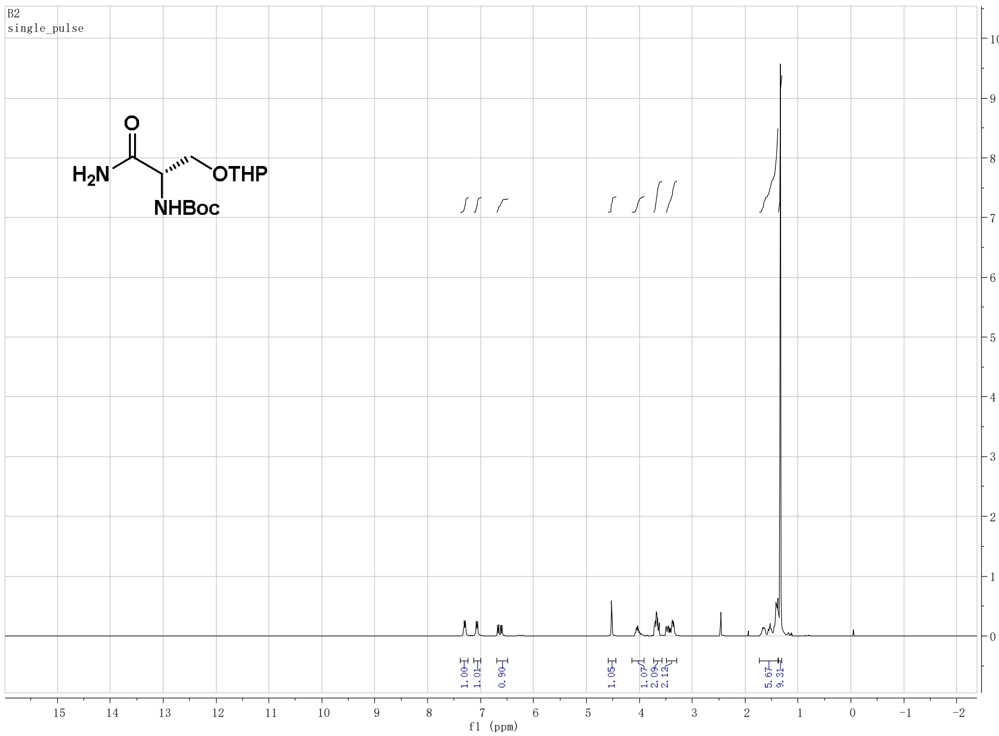

Supplementary Figure S1 ^{1}H-NMR spectrum of 7

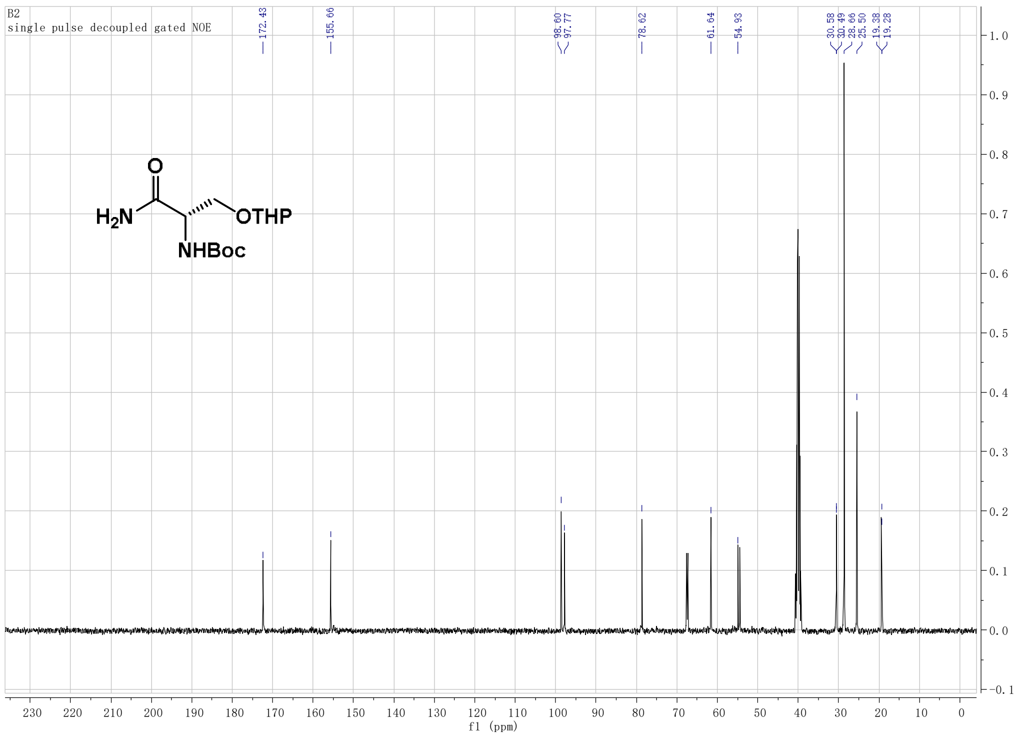

Supplementary Figure S2 ^{13}C-NMR spectrum of 7

Qualitative Analysis Report

**Data Filename**	1980.d	**Sample Name**	L-288
**Instrument Name**	TOF G6230A	**Acquired Time**	2023-04-06
**Acq Method**	YCL.M	**Acquired SW**	6200 series TOF/6500 series
**IRM Calibration Status**	**Success**		
**User Chormatograms**			

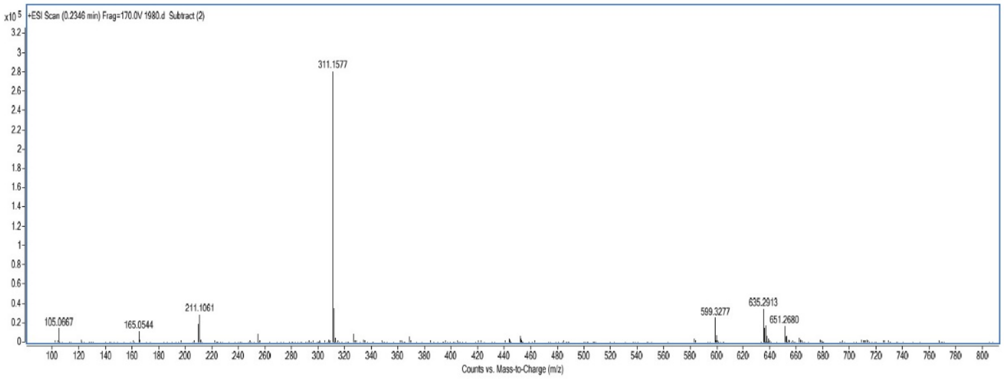

Supplementary Figure S3 HR-ESI-MS spectrum of 7

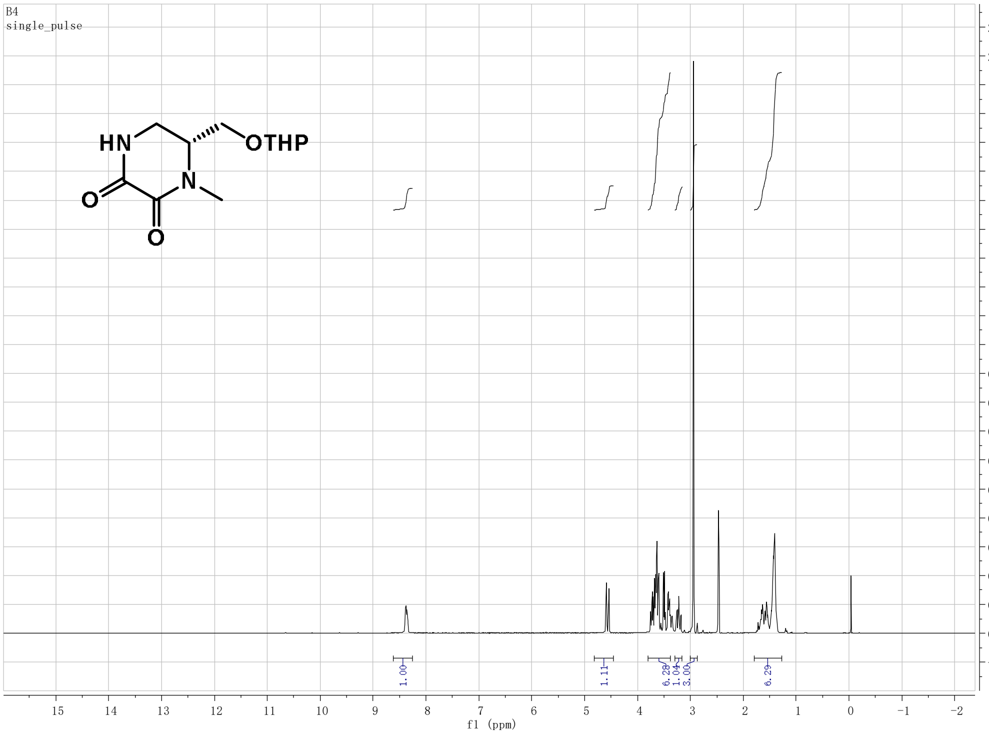

Supplementary Figure S4 ^{1}H-NMR spectrum of 9

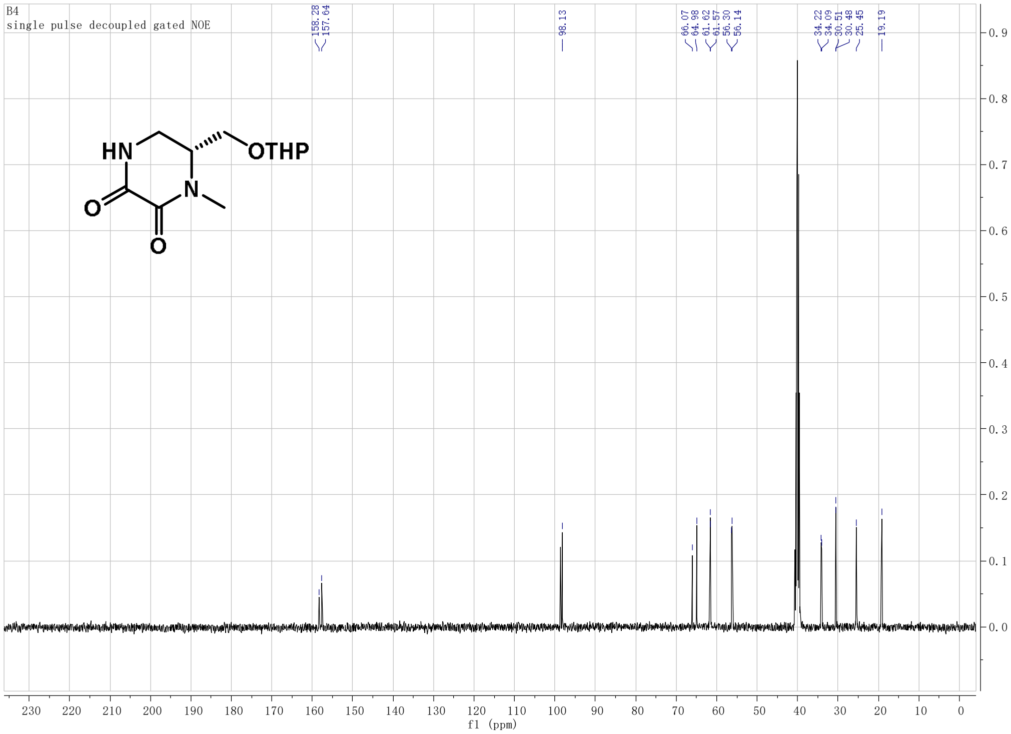

Supplementary Figure S5 ^{13}C-NMR spectrum of 9

Supplementary Figure S6 HR-ESI-MS spectrum of 9

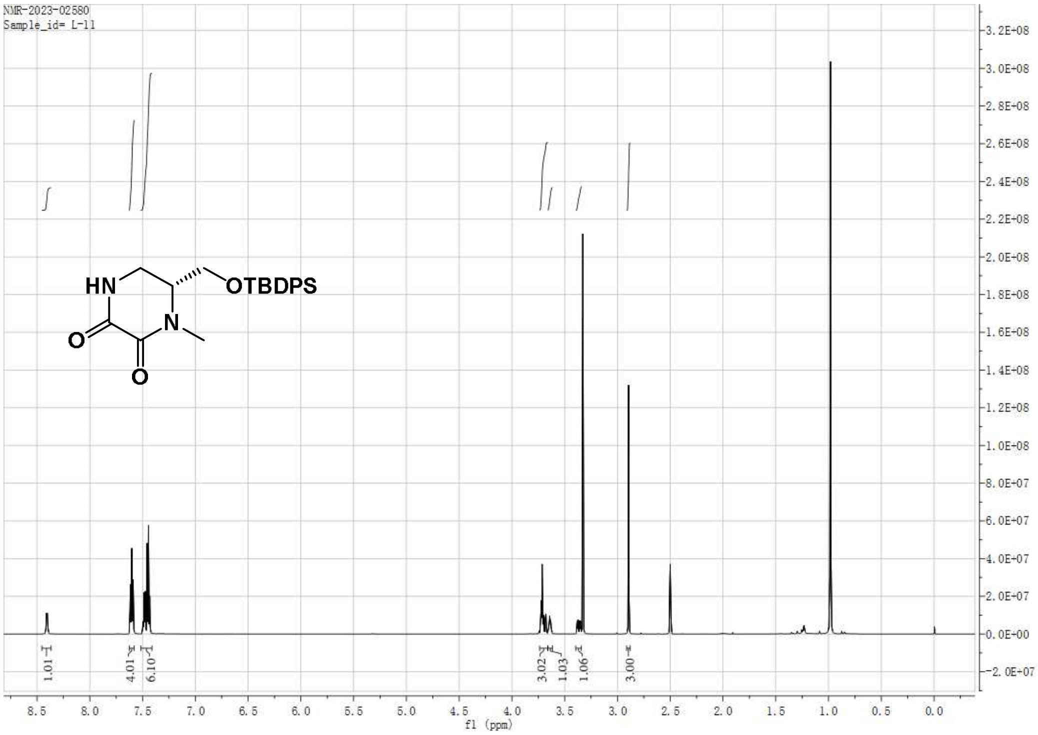

Supplementary Figure S7 ^{1}H-NMR spectrum of 11

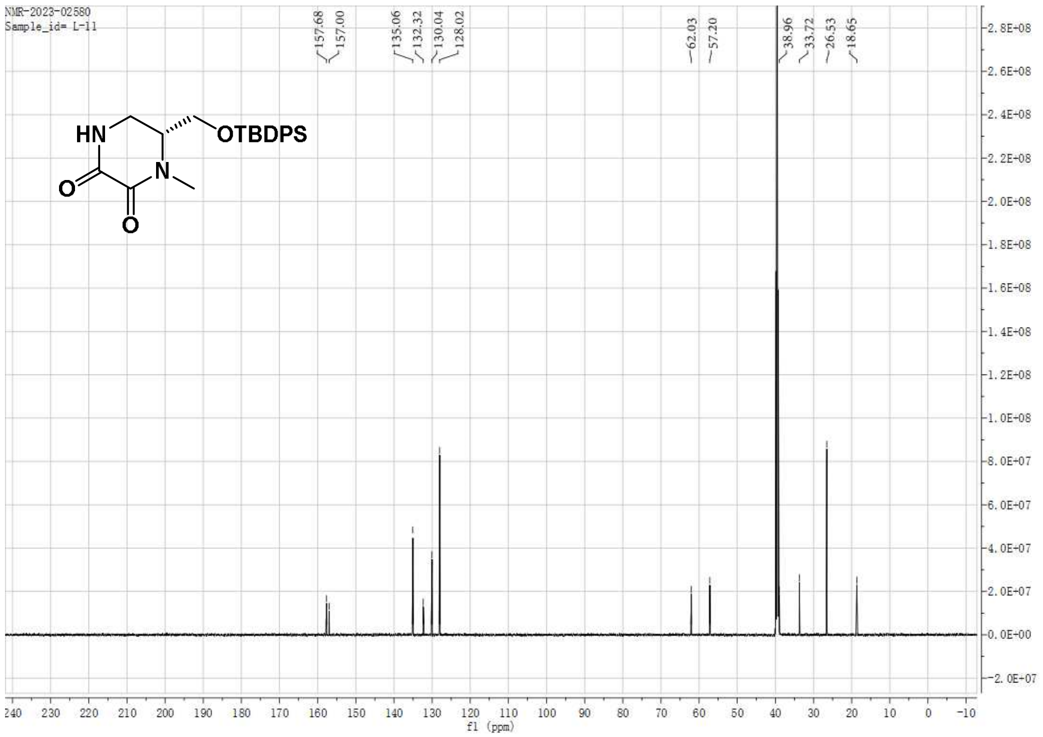

Supplementary Figure S8 ^{13}C-NMR spectrum of 11

Qualitative Analysis Report

**Data Filename**	1977.d	**Sample Name**	L-396
**Instrument Name**	TOF G6230A	**Acquired Time**	2023-04-06
**Acq Method**	YCL.M	**Acquired SW**	6200 series TOF/6500 series
**IRM Calibration Status**	**Success**		
**User Chormatograms**			

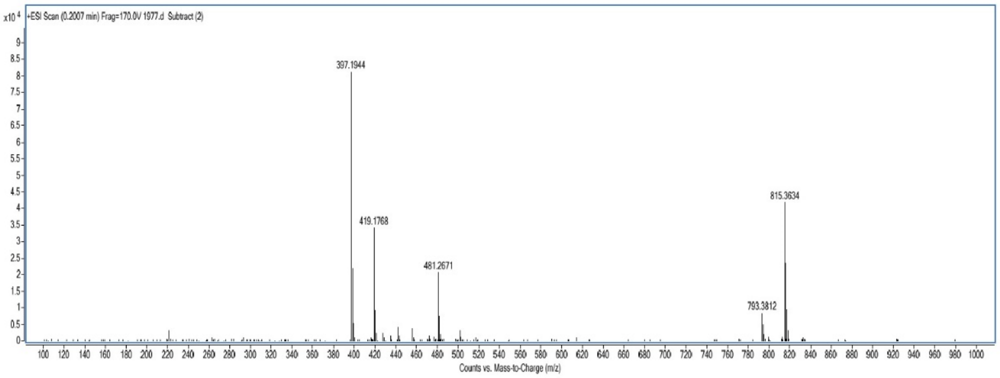

Supplementary Figure S9 HR-ESI-MS spectrum of 11

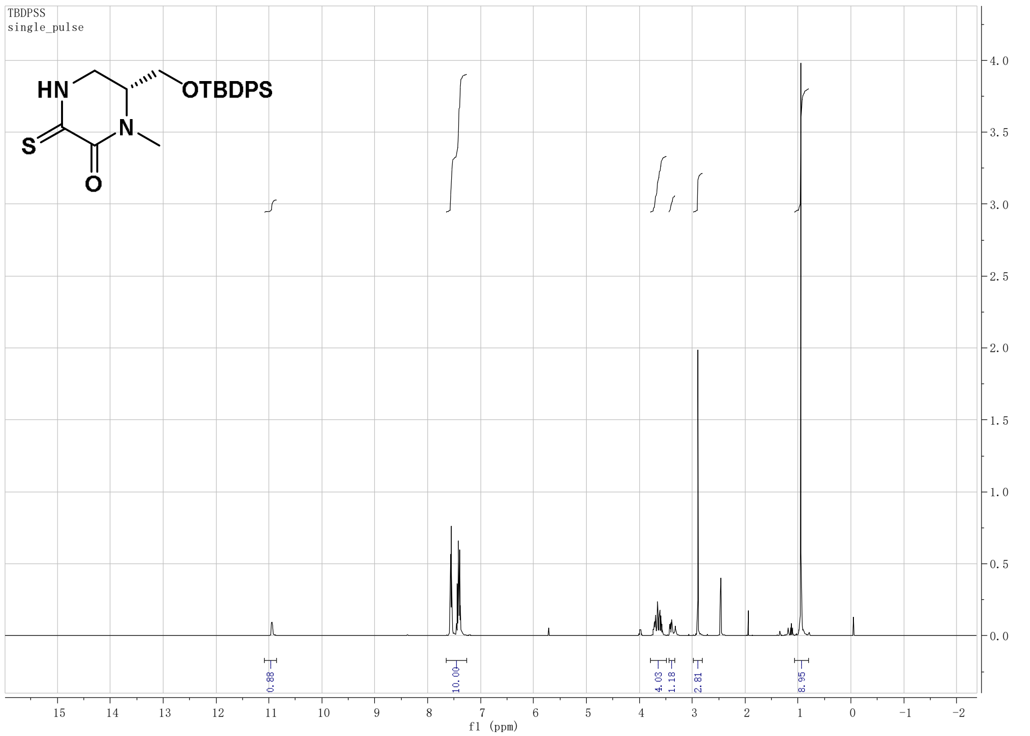

Supplementary Figure S10 ^{1}H-NMR spectrum of 12

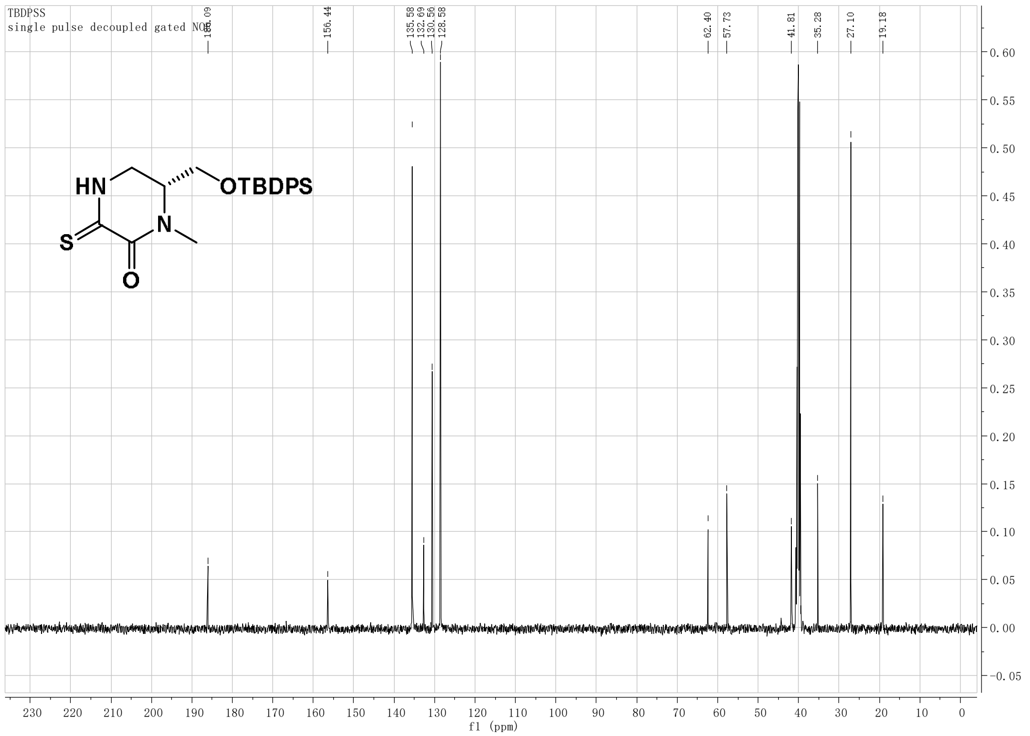

Supplementary Figure S11 ^{13}C-NMR spectrum of 12

Qualitative Analysis Report

Data Filename	1978.d	Sample Name	L-412
Instrument Name	TOF G6230A	Acquired Time	2023-04-06
Acq Method	YCL.M	Acquired SW	6200 series TOF/6500 series
IRM Calibration Status	Success		
User Chormatograms			

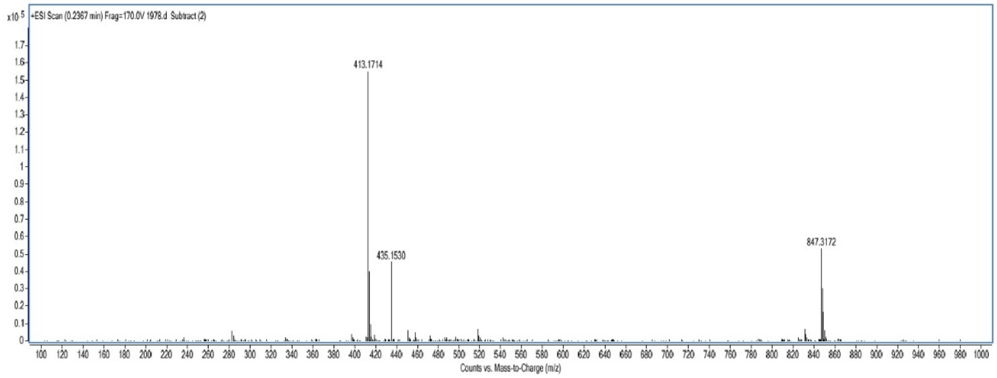

Supplementary Figure S12 HR-ESI-MS spectrum of 12

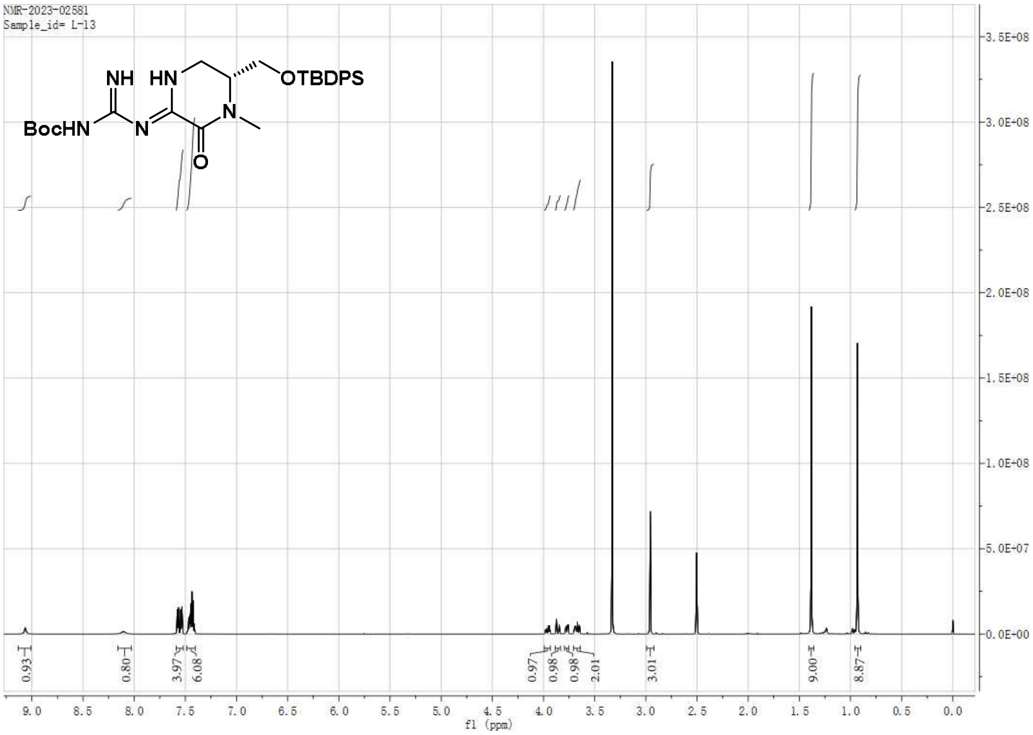

Supplementary Figure S13 ^{1}H-NMR spectrum of 13

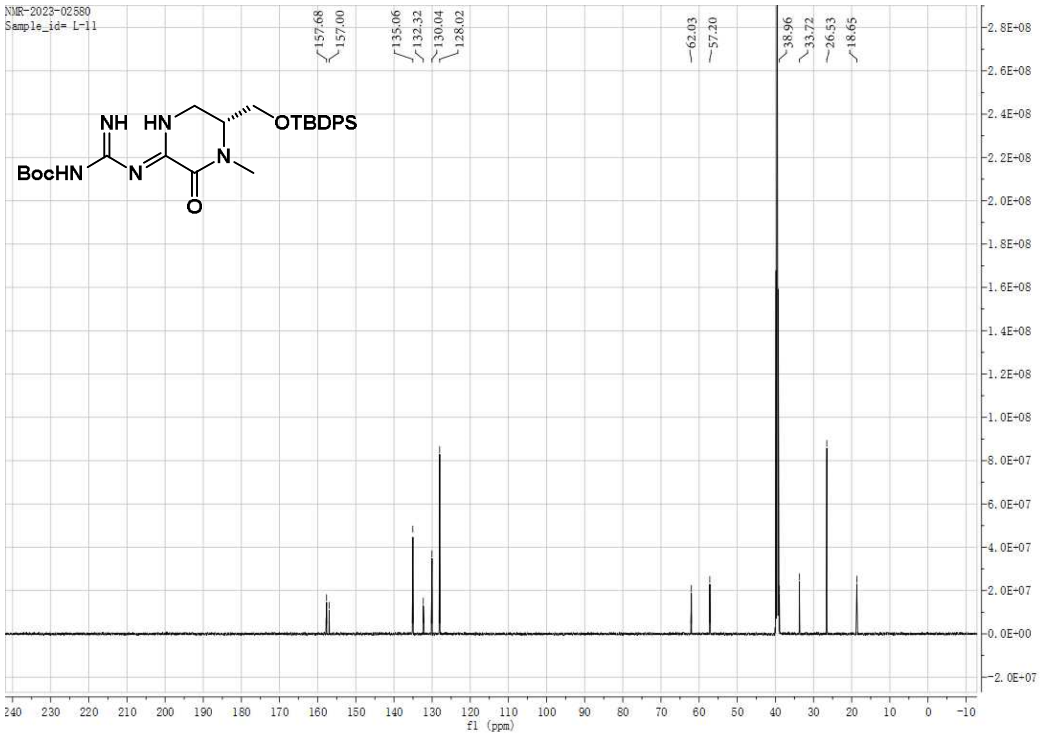

Supplementary Figure S14 ^{13}C-NMR spectrum of 13

Qualitative Analysis Report

**Data Filename**	2025.d	**Sample Name**	L-537
**Instrument Name**	TOF G6230A	**Acquired Time**	2023-04-07
**Acq Method**	YCL.M	**Acquired SW**	6200 series TOF/6500 series
**IRM Calibration Status**	**Success**		
**User Chormatograms**			

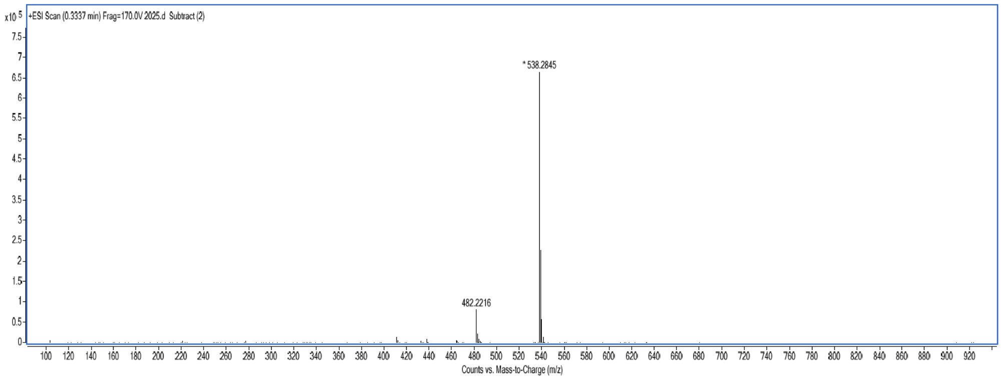

Supplementary Figure S15 HR-ESI-MS spectrum of 13

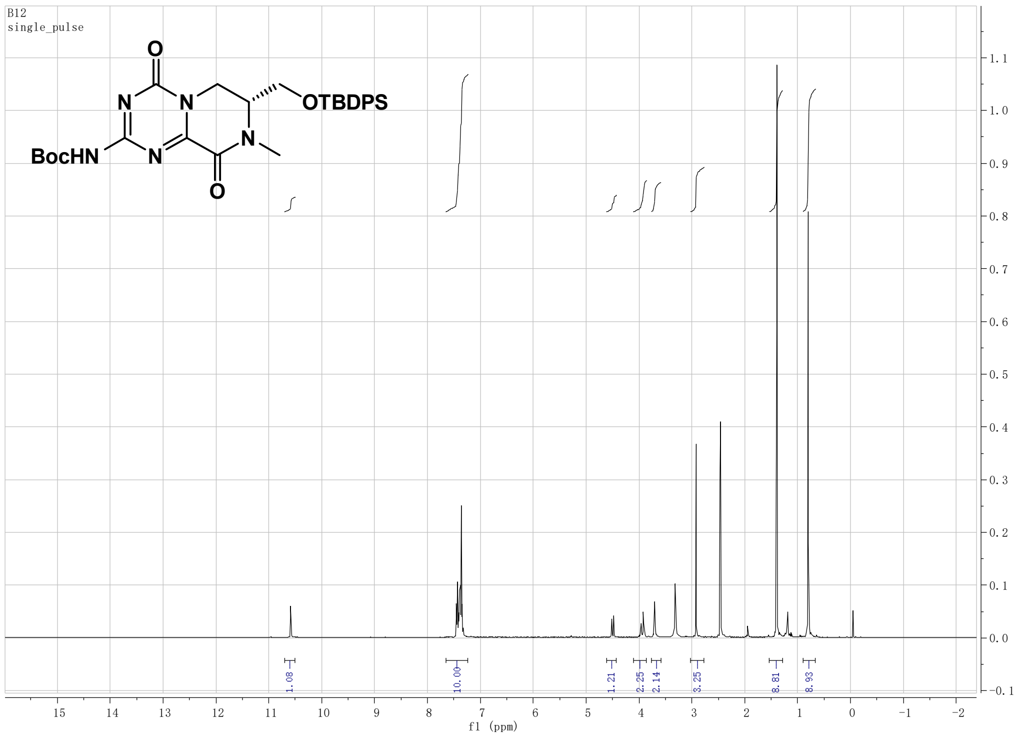

Supplementary Figure S16 ^{1}H-NMR spectrum of 14

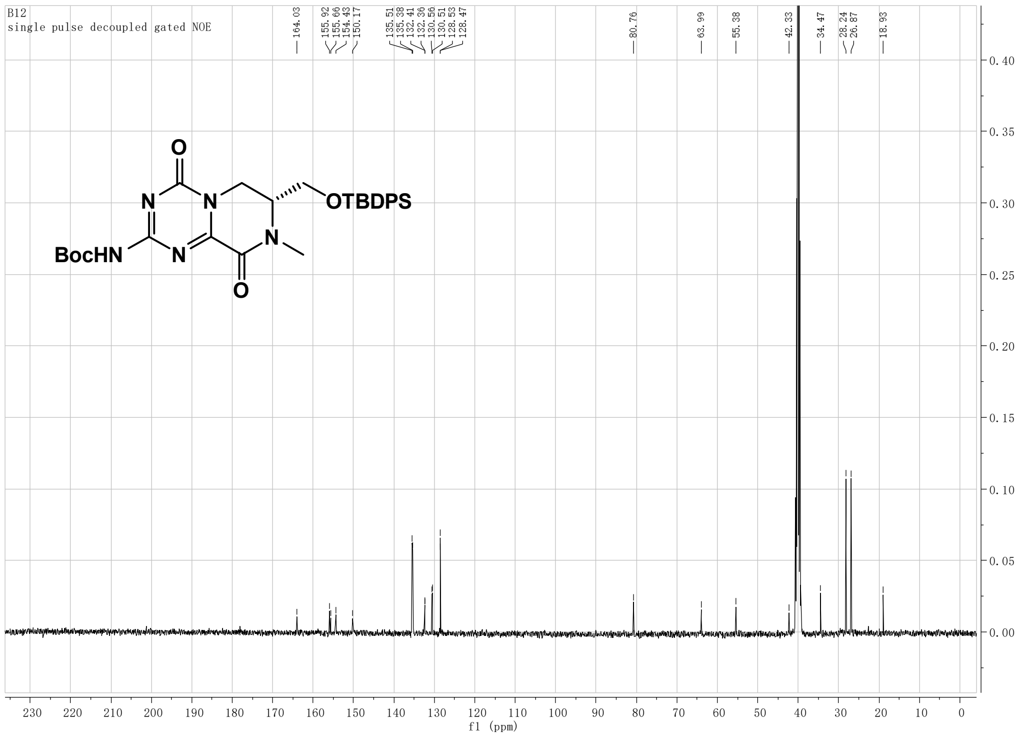

Supplementary Figure S17 ^{13}C-NMR spectrum of 14

Qualitative Analysis Report

**Data Filename**	2026.d	**Sample Name**	L-563
**Instrument Name**	TOF G6230A	**Acquired Time**	2023-04-07
**Acq Method**	YCL.M	**Acquired SW**	6200 series TOF/6500 series
**IRM Calibration Status**	Success		
**User Chormatograms**			

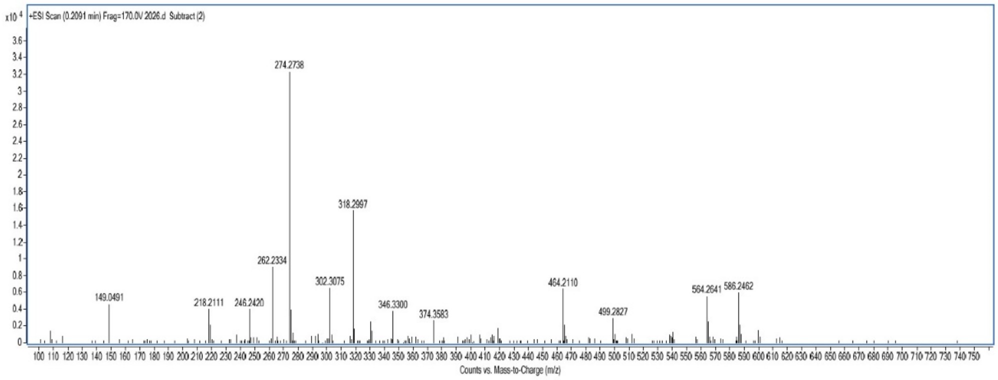

Supplementary Figure S18 HR-ESI-MS spectrum of 14

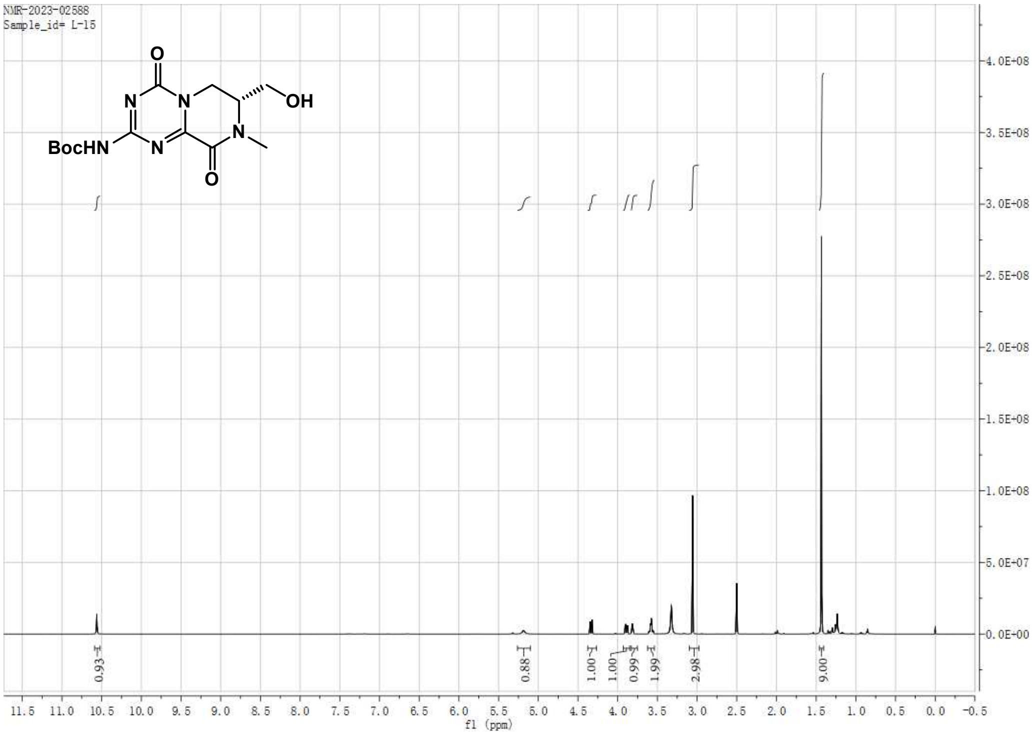

Supplementary Figure S19 ^{1}H-NMR spectrum of 15

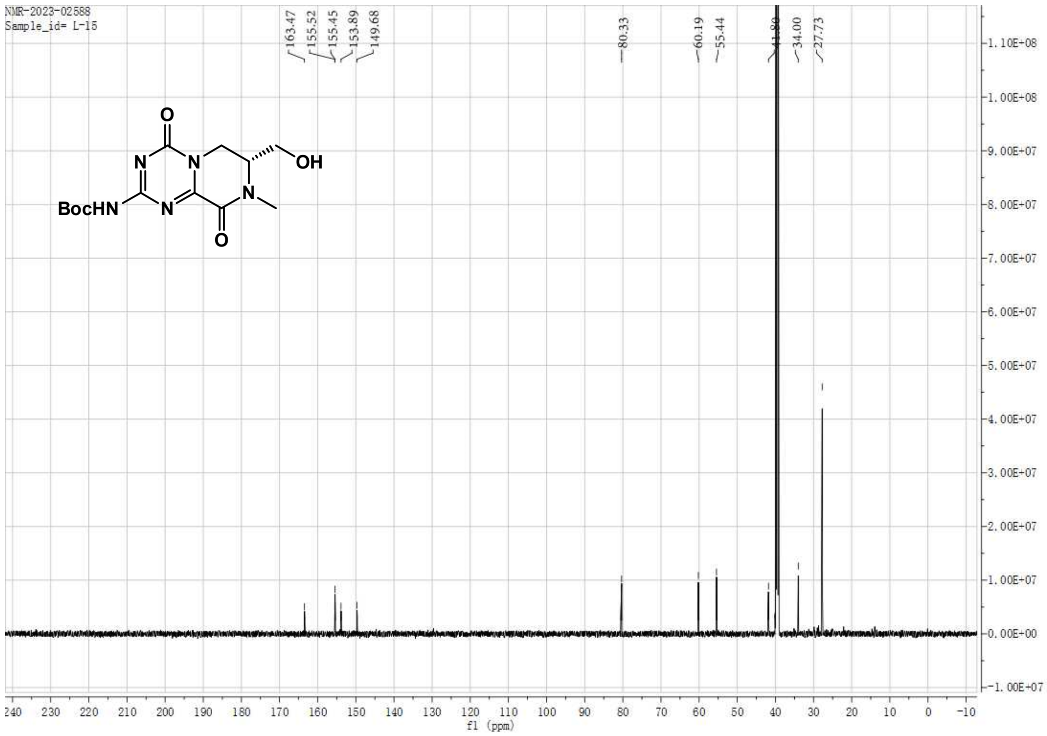

Supplementary Figure S20 ^{13}C-NMR spectrum of 15

Qualitative Analysis Report

**Data Filename**	1979.d	**Sample Name**	L-325
**Instrument Name**	TOF G6230A	**Acquired Time**	2023-04-06
**Acq Method**	YCL.M	**Acquired SW**	6200 series TOF/6500 series
**IRM Calibration Status**	**Success**		
**User Chormatograms**			

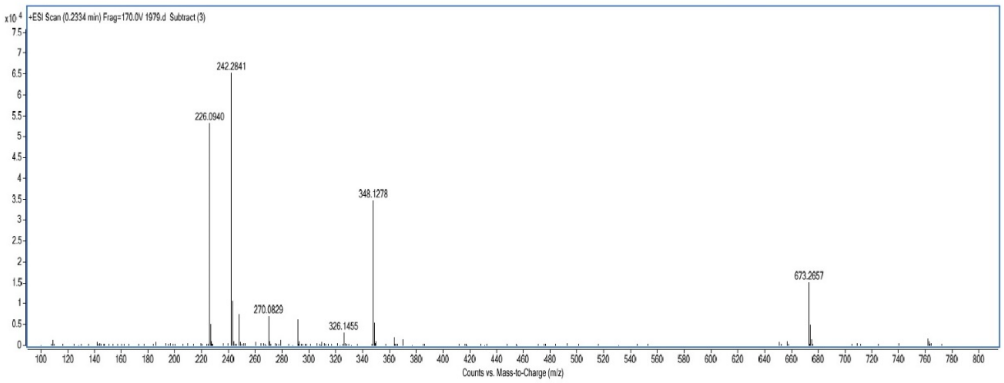

Supplementary Figure S21 HR-ESI-MS spectrum of 15

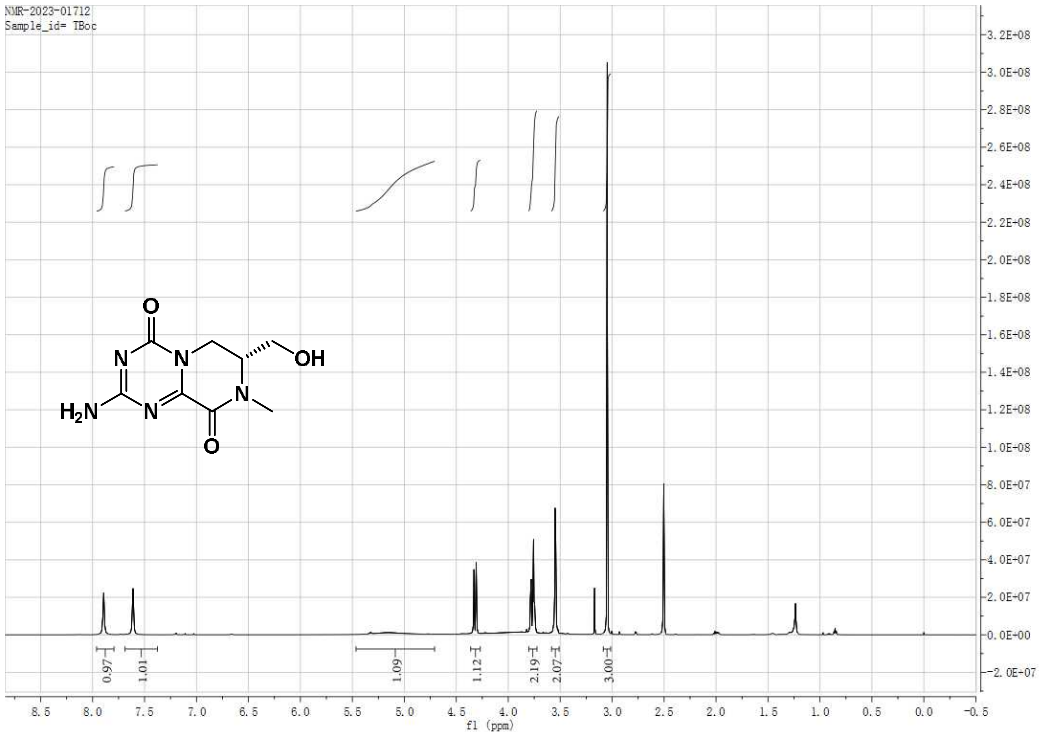

Supplementary Figure S22 ^{1}H-NMR spectrum of 1

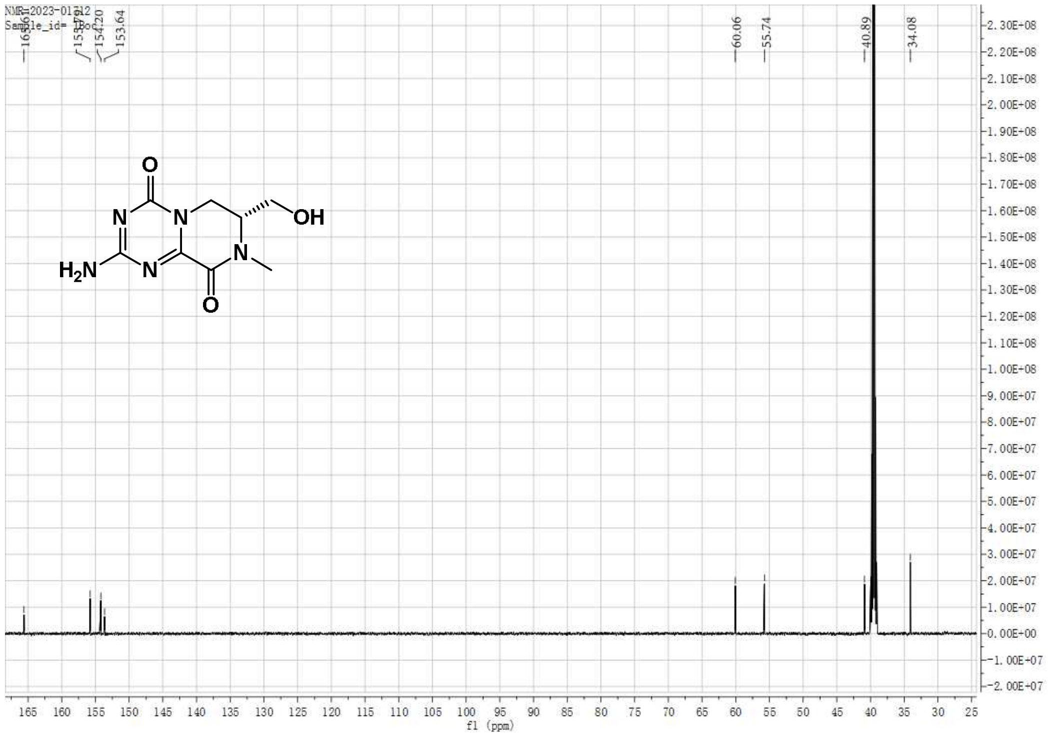

Supplementary Figure S23 ^{13}C-NMR spectrum of 1

Qualitative Analysis Report

**Data Filename**	1624.d	**Sample Name**	TBoc
**Instrument Name**	TOF G6230A	**Acquired Time**	2023-03-23
**Acq Method**	YCL.M	**Acquired SW**	6200 series TOF/6500 series
**IRM Calibration Status**	**Success**		
**User Chormatograms**			

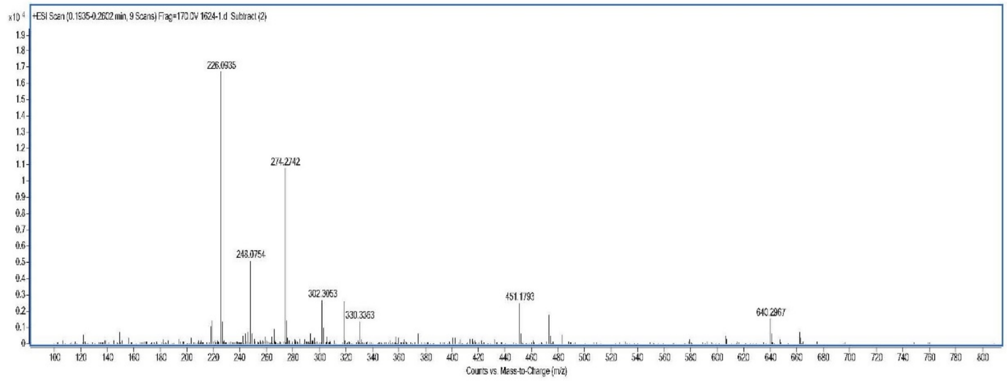

Supplementary Figure S24 HR-ESI-MS spectrum of 1

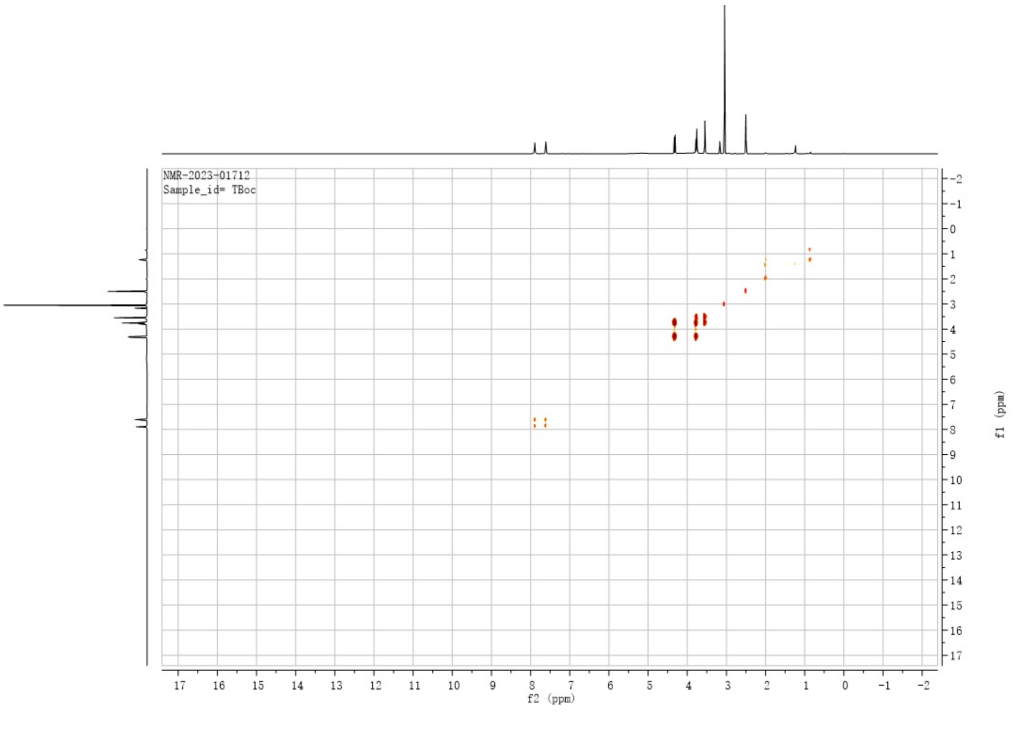

Supplementary Figure S25 ^{1}H -^{1}H COSY spectrum of 1

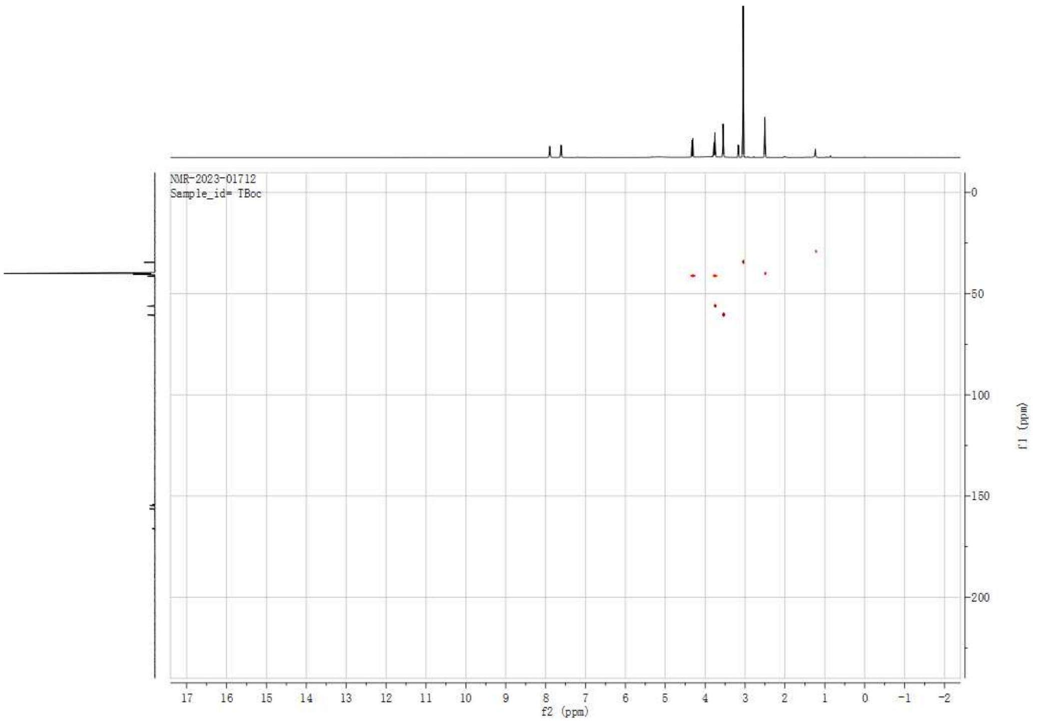

Supplementary Figure S26 HSQC spectrum of 1

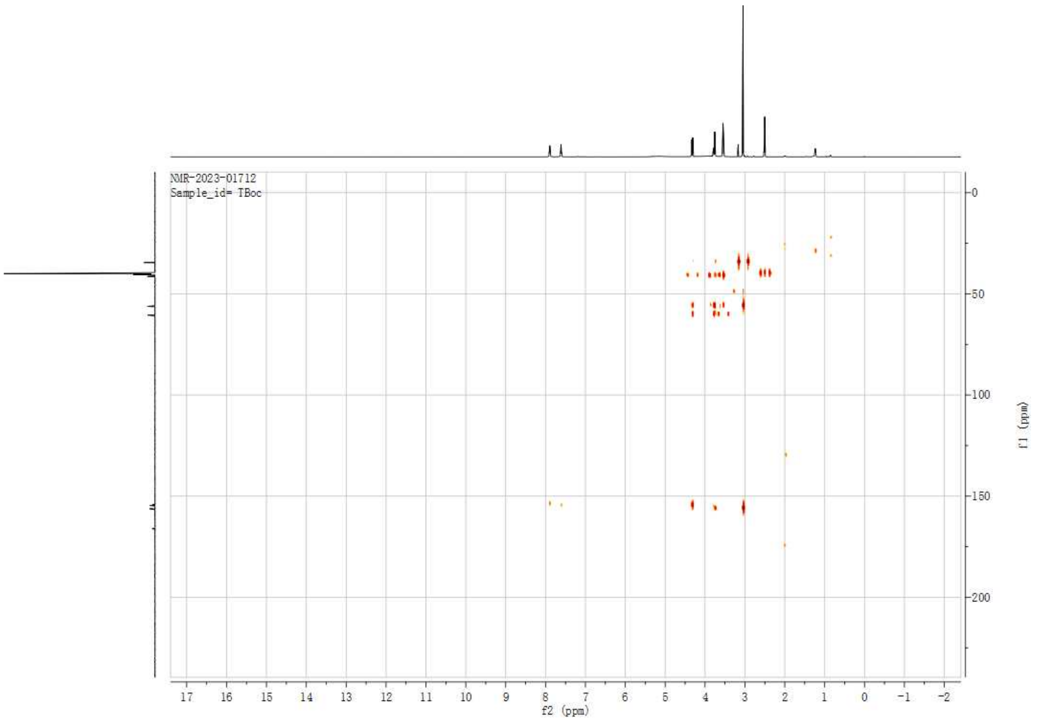

Supplementary Figure S27 HMBC spectrum of 1

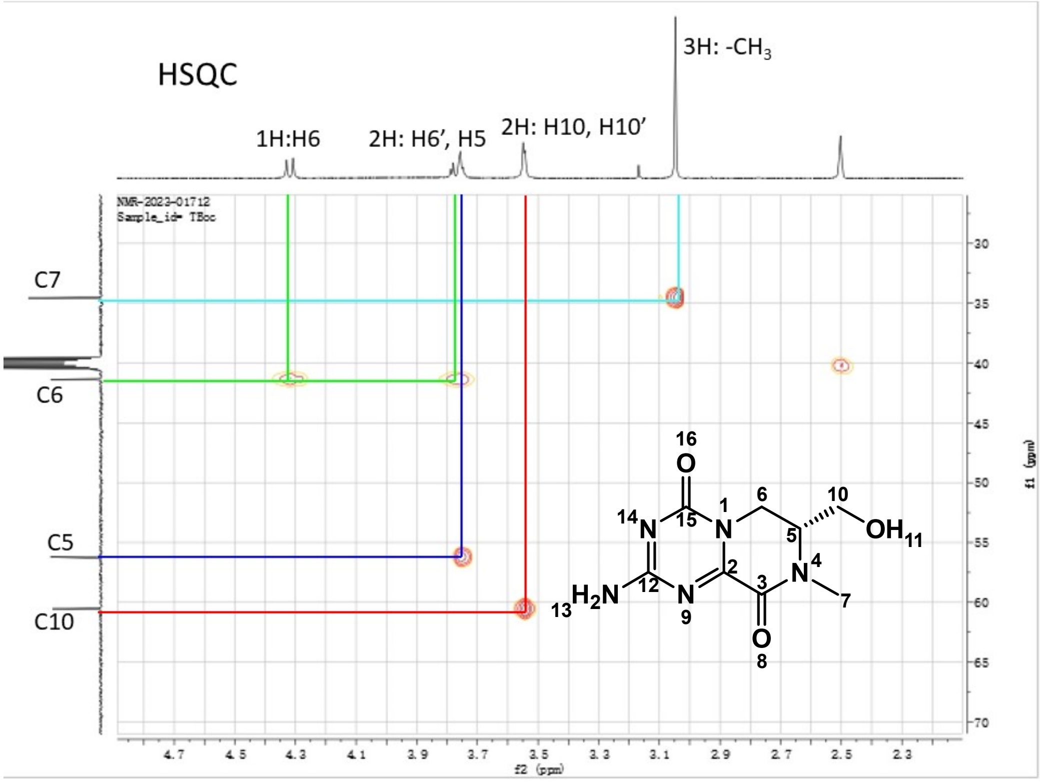
HSQC
1H:H6
2H: H6′, H5
2H: H10, H10′
3H: -CH3
NMR-2023-01712
Sample_id= TBoc
C7
C6
C5
C10
f2 (ppm)
f1 (ppm)

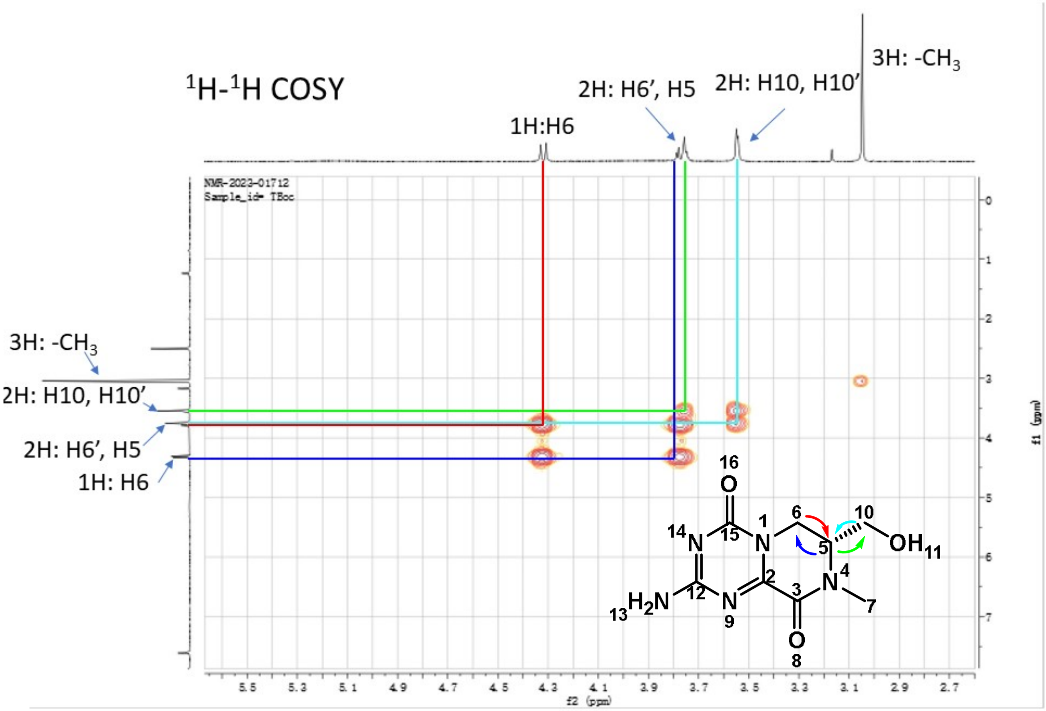
1H-1H COSY
1H:H6
2H: H6′, H5
2H: H10, H10′
3H: -CH3
NMR-2023-01712
Sample_id= TBoc
3H: -CH3
2H: H10, H10′
2H: H6′, H5
1H: H6
f2 (ppm)
f1 (ppm)

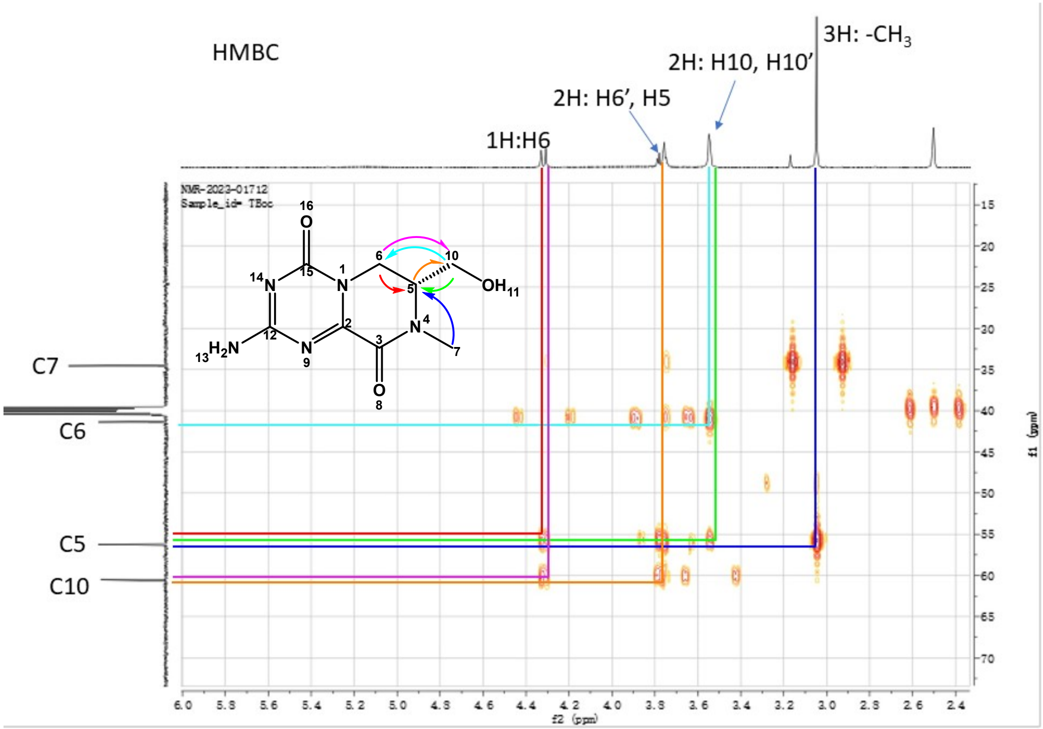
HMBC
1H:H6
2H: H6', H5
2H: H10, H10'
3H: -CH$_3$
C7
C6
C5
C10
NMR-2023-01712
Sample_id= TBoc
f2 (ppm)
f1 (ppm)

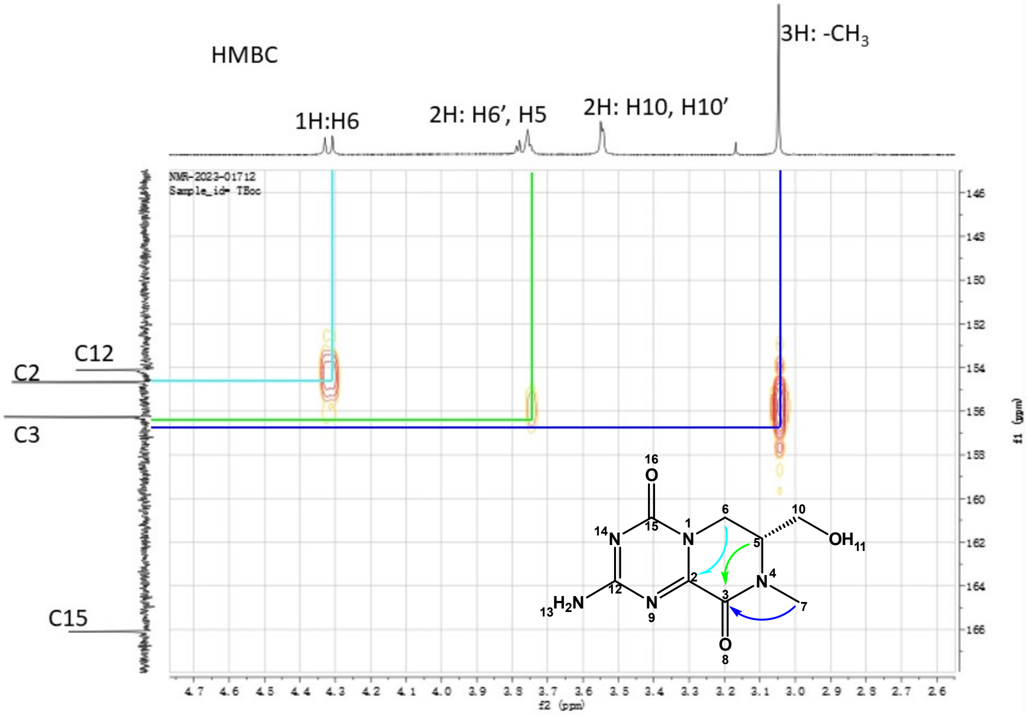
HMBC
1H:H6
2H: H6', H5
2H: H10, H10'
3H: -CH$_3$
C12
C2
C3
C15
NMR-2023-01712
Sample_id= TBoc
f2 (ppm)
f1 (ppm)
